# Supplementary material for: Boosting Reservoir Computing with Brain-inspired Adaptive Dynamics
Source: ArXiv. 2025 Apr 16:arXiv:2504.12480v1. Preprint. [Version 1] (PMC12047930)
Supplement: Supplement 1 [file NIHPP2504.12480v1-supplement-1.pdf]

Supplementary Material
Boosting Reservoir Computing with Brain-inspired Adaptive Dynamics

Keshav Srinivasan,[1,2] Dietmar Plenz,[2] and Michelle Girvan[1,3,4]

[1] *Biophysics Program, University of Maryland, College Park, MD 20740, USA*
[2] *Section on Critical Brain Dynamics, National Institute of Mental Health, Bethesda, MD 20892, USA*
[3] *Department of Physics, University of Maryland, College Park, MD 20740, USA*
[4] *Santa Fe Institute, Santa Fe, NM 87501, USA*

arXiv:2504.12480v1 [cs.NE] 16 Apr 2025

SUPPLEMENTARY NOTE: CALCULATION FOR 'DESIGNED' RESERVOIRS

Consider the 2 reservoir equations (refer to Equations 1 and 2 from the main text):

$$V_i(t+1) = \lambda_i V_i(t) + \sum_{j=1}^{N} \left(A_{ij}^E - A_{ij}^I\right) \cdot r_j(t) + W_i^{in} u(t) \tag{1}$$

$$r_i\,(t) = \text{Sig}\,\left(V_i\,(t) - \theta_i\,(t)\right) \tag{2}$$

As mentioned in the main text, here $V_i(t)$ is the membrane potential of the ith neuron, λ is the leakage term (which in this case is set to 0), A is the connectivity matrix of the reservoir network, W^{in} is the input matrix and $u(t)$ is the input data. The second equation defines the reservoir variable, $r(t)$, which represents the firing rate of the neurons in the network and can take values between 0 and 1. Sig is a sigmoid function and is given the following equation:

$$\text{Sig}(y) = \frac{1}{1 + e^{-10y}} \tag{3}$$

Consider a steady-state solution with a firing rate set-point of ρ. We can now rewrite the steady-state version of Equation (1) as:

$$\widetilde{V}_i = \sum_{j=1}^{N} \widetilde{A}_{ij} \rho_j + W_i^{in} \langle u \rangle \tag{4}$$

Inverting equation 2 for the steady state solution, we also obtain:

$$\widetilde{V}_i = \text{Logit}(\rho_i) + \theta_i \tag{5}$$

Here Logit is the inverse Sigmoid function. Combining equations 4 and 5 we then get:

$$\sum_{j=1}^{N} \widetilde{A}_{ij} \rho_j = \text{Logit}(\rho_i) + \theta_i - W_i^{in} \langle u \rangle \tag{6}$$

Let us consider a design mechanism that only alters the inhibitory links by multiplying them by a neuron-wise multiplicative factor, Ω_i.

Before the adjustment, we have the following equation (Split into excitatory and inhibitory sub-parts):

$$\sum_{j=1}^{N} A_{ij} \rho_j = \sum_{E} A_{ij} \rho_j + \sum_{I} A_{ij} \rho_j \tag{7}$$

And after the adjustment, we get the following equation:

$$\sum_{j=1}^{N} \widetilde{A}_{ij} \rho_j = \sum_{E} A_{ij} \rho_j + \Omega_i \sum_{I} A_{ij} \rho_j \tag{8}$$

Combining Eqns 6 and 8 we then finally get an expression for the multiplicative factor, Ω_i:

$$\boxed{\Omega_i = \frac{\text{Logit}(\rho_i) + \theta_i - W_i^{in} \langle u \rangle - \sum_E A_{ij} \rho_j}{\sum_I A_{ij} \rho_j}} \tag{9}$$

SUPPLEMENTARY FIGURES

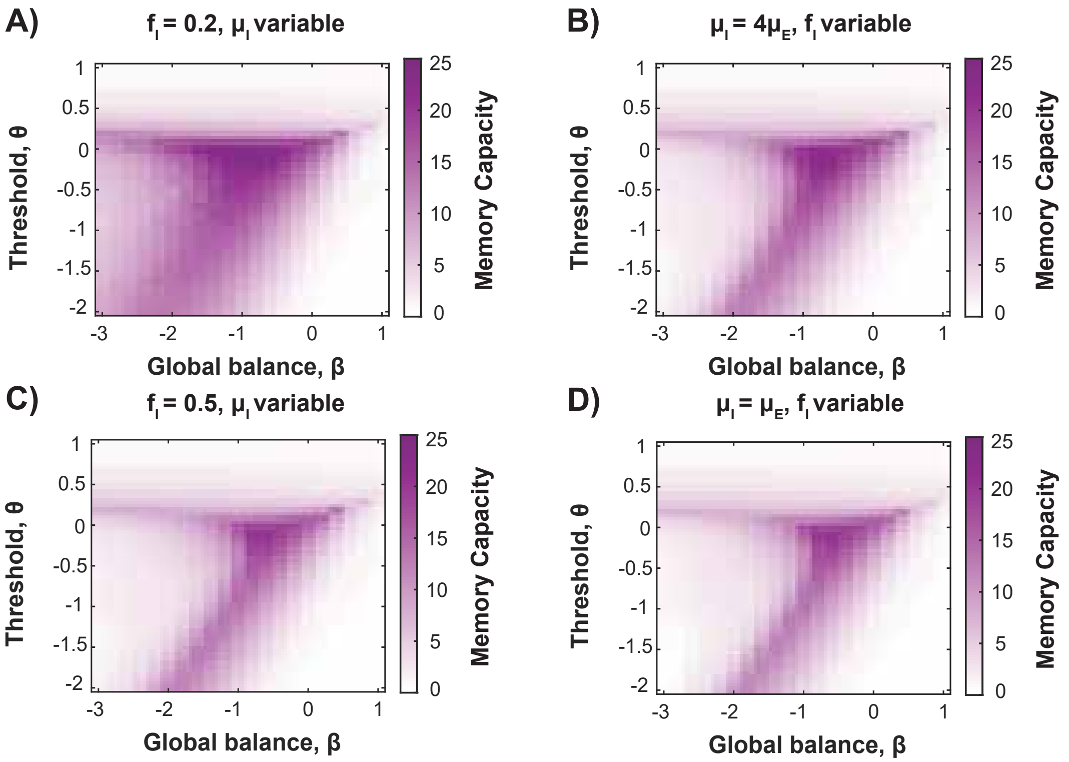

Supplementary Figure 1. **Reservoir performance remains robust across different forms of E-I balance modulation.** Memory capacity is shown as a function of threshold (θ) and global balance (β). We vary global balance in two ways: by changing the mean strength of inhibitory links (**A, C**) and by changing the fraction of inhibitory neurons (**B, D**). In (**A**) and (**C**), the proportion of inhibitory neurons is fixed (20% and 50%, respectively), In (**B**) and (**D**), the E/I synaptic strength ratio is fixed (1:4 and 1:1, respectively). Across all conditions, high performance is consistently observed in balanced to slightly inhibited regimes, demonstrating the robustness of reservoir dynamics to the method of E-I tuning.

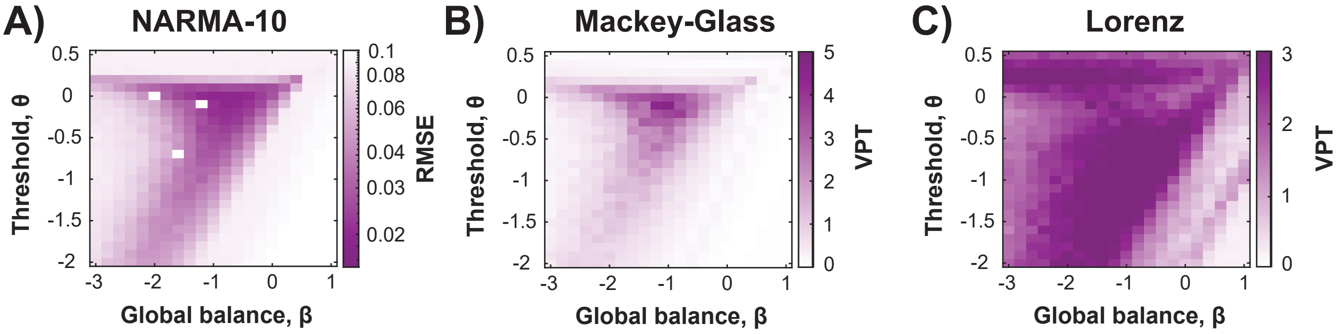

Supplementary Figure 2. **Performance as a function of the balance parameter β and threshold θ for three time-series prediction tasks: NARMA-10, Mackey-Glass, and Lorenz.** This figure expands on Fig. 1C in the main text, offering a detailed analysis of performance trends across these tasks. Performance remains high and stable in balanced or slightly over-inhibited states but declines sharply in the over-excited regime, where it becomes more fragile across all three tasks.

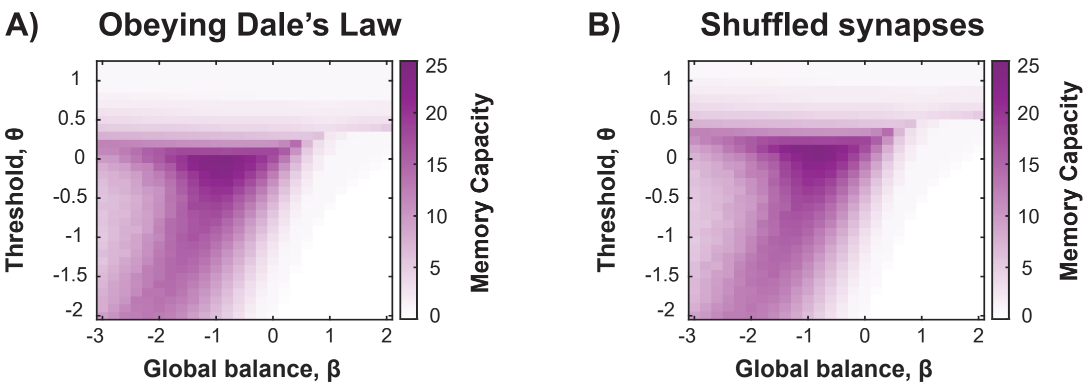

Supplementary Figure 3. **Reservoir dynamics and performance remains consistent despite synapse shuffling, indicating no strong dependence on Dale's Law.** (**A**) A globally tuned excitatory-inhibitory (E-I) reservoir where neurons and synapses strictly follow Dale's Law (as shown in Fig. 1D). (**B**) A modified reservoir where synapses are randomly shuffled, disrupting Dale's Law while preserving overall connectivity statistics. Despite this alteration, performance remains unchanged across different values of the global balance parameter (β) and threshold (θ), demonstrating that adherence to Dale's Law is not a necessary condition for RC performance

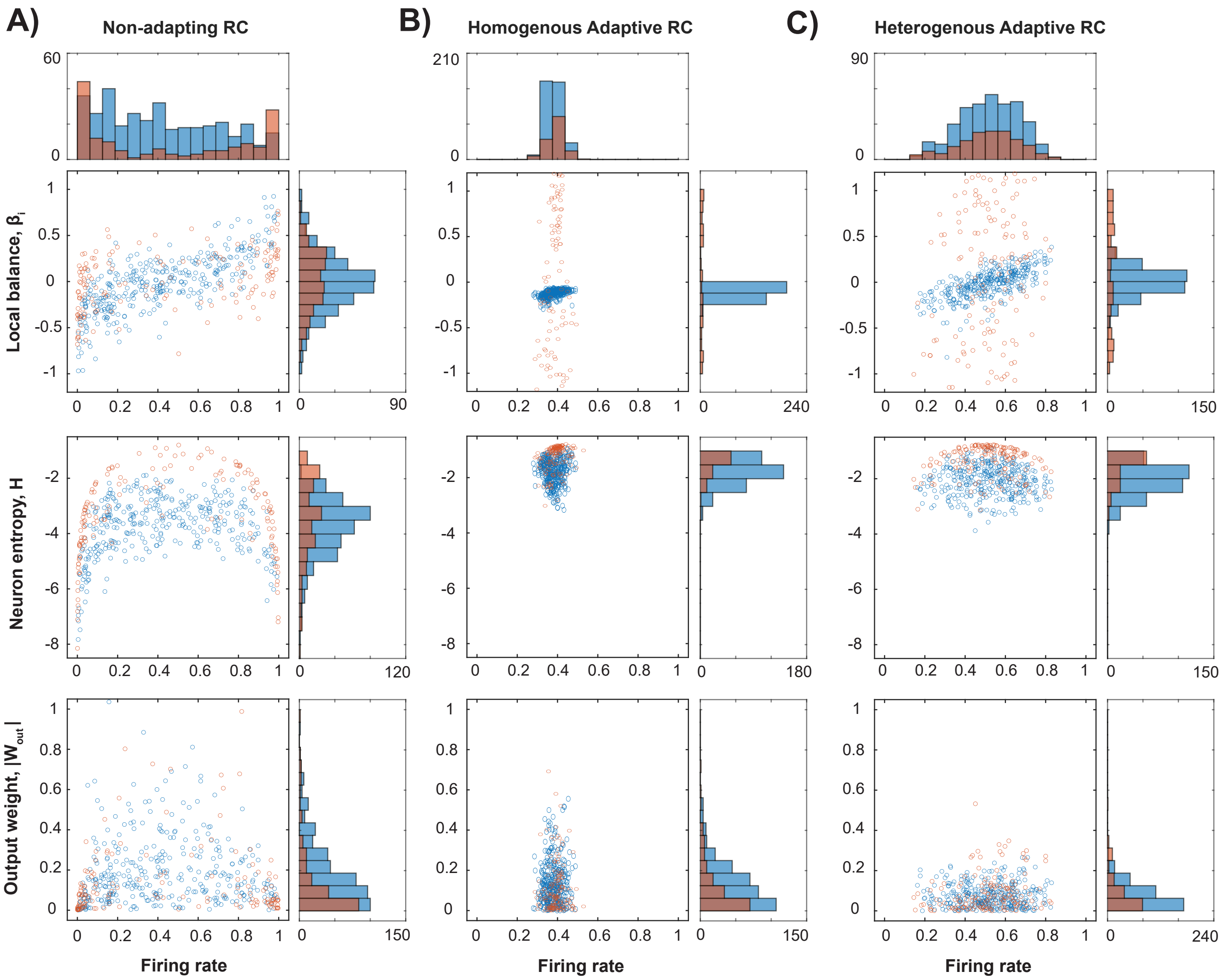

Supplementary Figure 4. **Scatter plots depicting the relationship between local balance, neuronal entropy, and output weights as functions of average firing rate for input and non-input neurons during a Lorenz time-series prediction task.** (**A**) In the scatter plots of a globally balanced, non-adapting reservoir computer (RC) ($\beta = 0$), we observe (*top*) that input neurons (*orange*) are driven more strongly, resulting in higher firing rates compared to non-input neurons (*blue*). (*middle*) The neurons with extreme firing rates show reduced entropy, which (*bottom*) leads to their diminished contribution to prediction accuracy. (**B**) When an adaptive RC with a homogeneous firing rate target of 0.4 is employed, (*top*) the adaptation mechanism tunes the network to achieve the desired firing rate. Here, input neurons exhibit substantial local imbalance to compensate for the strong input signals. (*middle*) This adaptation process successfully eliminates low-entropy neurons, (bottom) ensuring that all neurons contribute adequately to predictions. (**C**) As in the previous case, with heterogeneous targets, (*top*) input neurons require significant local imbalance to adjust for the strong input. By mitigating extreme firing rates, (*middle*) we also eliminate low-entropy neurons, (*bottom*) resulting in a trend in output weights that parallels the findings with homogeneous targets.

SUPPLEMENTARY TABLE

**Task**	**Non-adaptive**			**Adaptive**		
				**Homogenous**		**Hetero.**
	σ_{in}	β	θ	σ_{in}	ρ_T	σ_{in}
Memory Capacity	0.016	-1.0	0.0	0.016	0.5	0.010
NARMA-10	0.100	-1.0	0.0	0.063	0.3	0.100
Mackey Glass	0.631	-1.2	0.0	0.631	0.3	1.000
Lorenz	2.512	-1.0	-1.0	3.981	0.6	3.981

Supplementary Table I. Task-dependent parameters used in the RC setup
